# Subcutaneous emphysema and pneumomediastinum following cocaine inhalation: a case report

**DOI:** 10.1186/s13256-015-0683-8

**Published:** 2015-09-13

**Authors:** Deanne S. Soares, Anna Ferdman, Rozanna Alli

**Affiliations:** Campbelltown Hospital, University of Western Sydney, Goldsmith Avenue, Campbelltown, NSW 2560 Australia

## Abstract

**Introduction:**

Subcutaneous emphysema or pneumomediastinum can occur as a complication of illicit drug use although this is rare. When occurring without a pneumothorax and spontaneously, it is usually treated conservatively, but can have serious consequences.

**Case presentation:**

Here, we present the case of an otherwise healthy 23-year-old Caucasian man who presented to the Emergency Department at our institution and was found to have both subcutaneous emphysema and pneumomediastinum as a result of cocaine use. His only presenting symptom was mild chest pain and he had palpable subcutaneous crepitations. He underwent a series of investigations including a chest radiograph and computed tomography as well as a barium fluoroscopy study to rule out secondary pneumomediastinum, which can be fatal. There were no other pulmonary features of illicit drug use, such as granulomas or fibrosis, seen on radiological imaging. He was subsequently managed with a period of observation and supportive care.

**Conclusion:**

We report a rare case of subcutaneous emphysema and pneumomediastinum likely due to the nasal insufflation of cocaine. We discuss the necessary investigations to rule out any serious underlying pathology. These should be considered in patients who present with chest pain after cocaine use.

## Introduction

Subcutaneous emphysema and pneumomediastinum are usually a consequence of esophageal or chest trauma, or are iatrogenic in nature. Causes include assisted ventilation and medical or dental procedures as well as several diseases. It can also spontaneously occur due to coughing, vomiting, and forceful straining, such as in childbirth or strenuous exercise [1]. However, subcutaneous emphysema and pneumomediastinum as a consequence of recreational drug use is very rare with only a few reported cases [2, 3]. Most of the previously reported cases of pneumomediastinum have occurred after cocaine was smoked; the fact that our patient developed pneumomediastinum after nasal insufflation of cocaine makes our case unusual [2, 3].

## Case presentation

An otherwise healthy 23-year-old Caucasian man presented to our Emergency Department reporting a sensation of “bubbles” under the skin of his neck and mild chest pain. He denied any dyspnea, neck pain, dysphagia, odynophagia, or dysphonia. He reported that he had “snorted” a small amount of cocaine two nights prior to his Emergency Department presentation. He did not have a history of chronic or regular drug use. There was no history of any trauma or vigorous physical exercise. He had mild rhinorrhea but no coughing or vomiting. He did not take any regular medication, but reported the occasional use of illicit drugs such as cocaine and ecstasy.

On examination, he was alert and responsive without any airway compromise or respiratory distress. His pulse rate was 96 beats per minute with a blood pressure of 120/73mmHg. He had a respiratory rate of 18 breaths per minute with oxygen saturation of 97% on room air and he was apyrexial. Subcutaneous crepitations were palpable across his neck and the superior part of his chest. The rest of his physical examination was unremarkable.

**Fig. 1:**
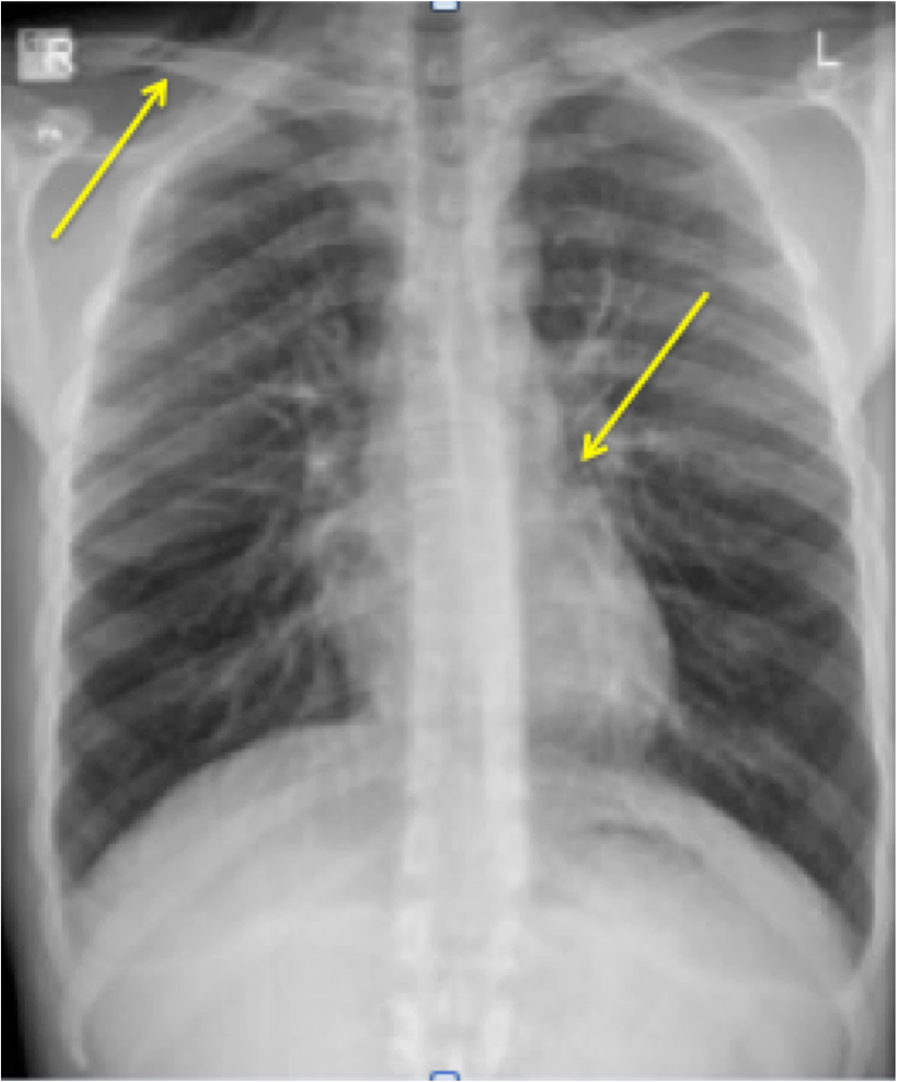
Plain posterioranterior chest radiograph showing pneumomediastinum and subcutaneous emphysema (bottom arrow and top arrow respectively)

**Fig. 2:**
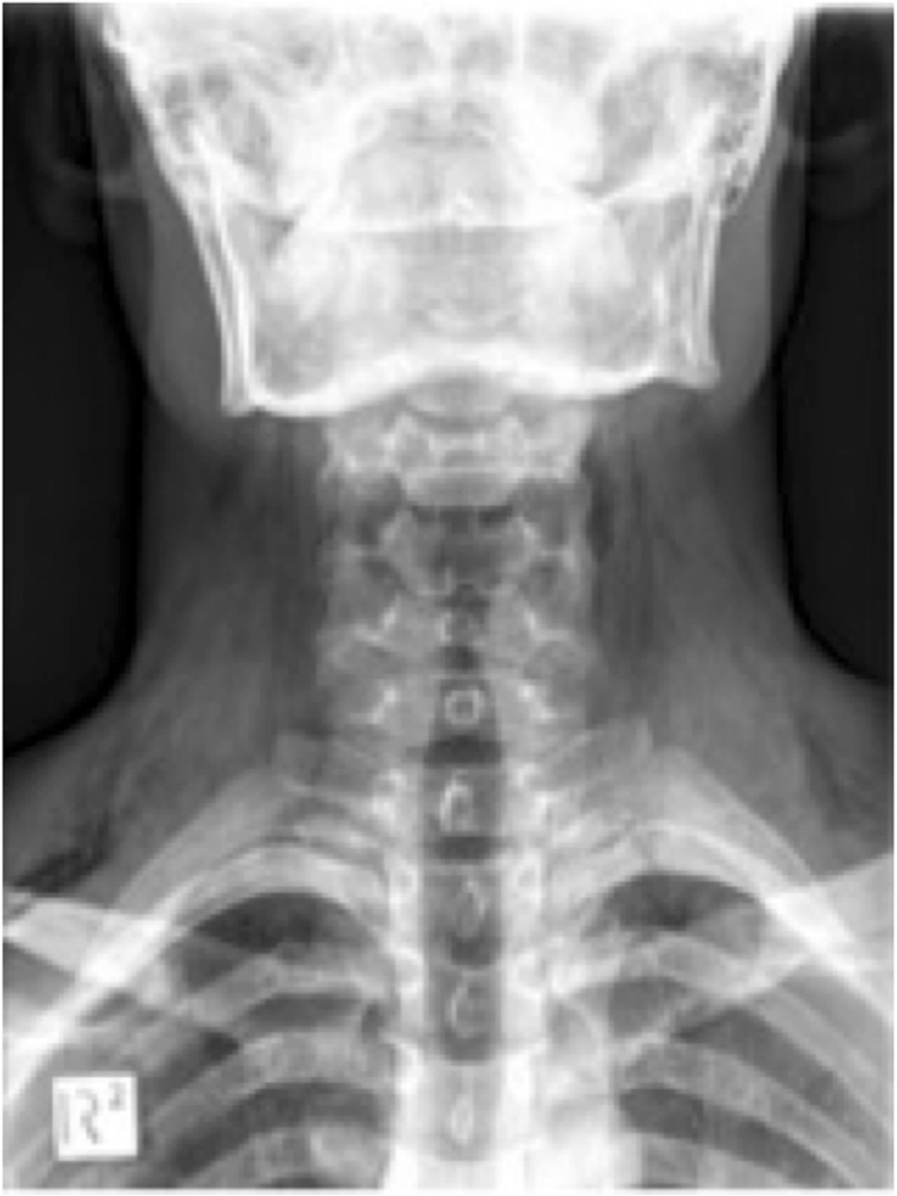
Plain radiograph of the neck showing extensive subcutaneous emphysema

**Fig. 3:**
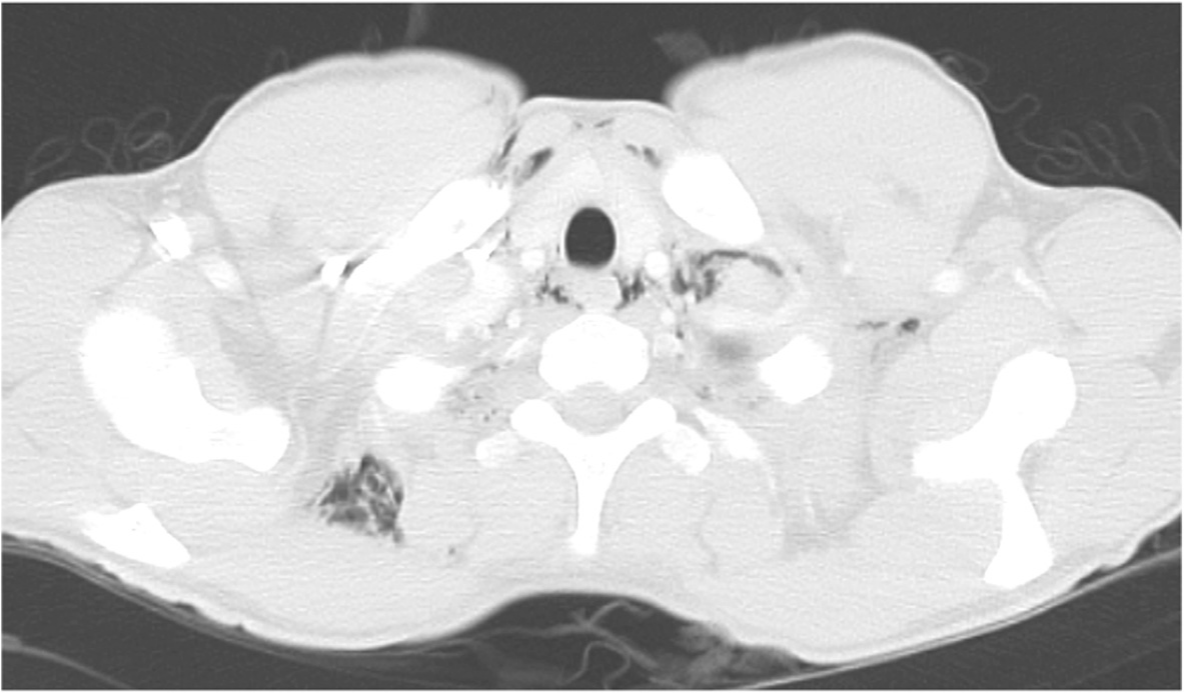
Axial computed tomography showing subcutaneous emphysema in the neck

**Fig. 4:**
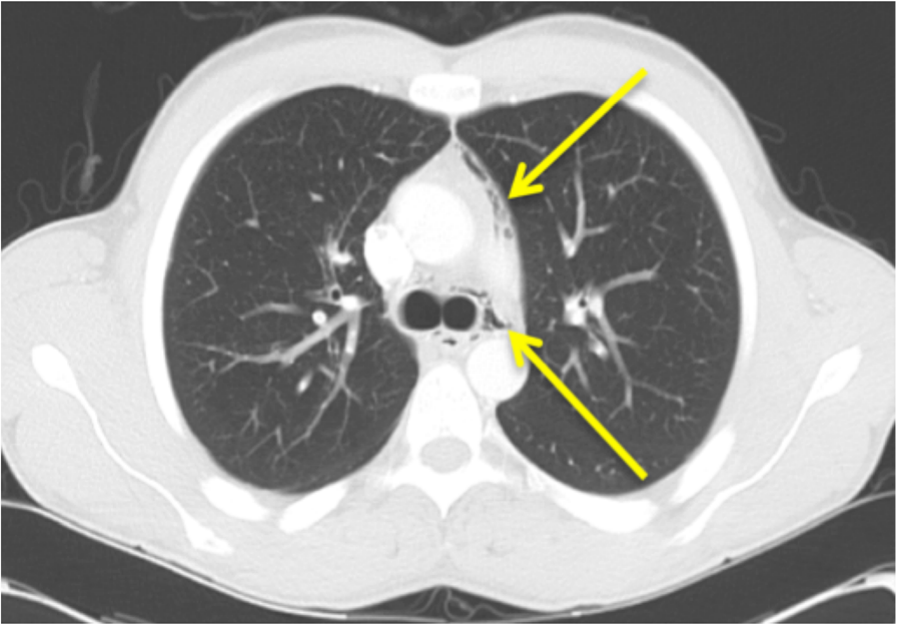
Axial chest computed tomography demonstrating multiple locules of gas within the mediastinum (both arrows)

**Fig. 5:**
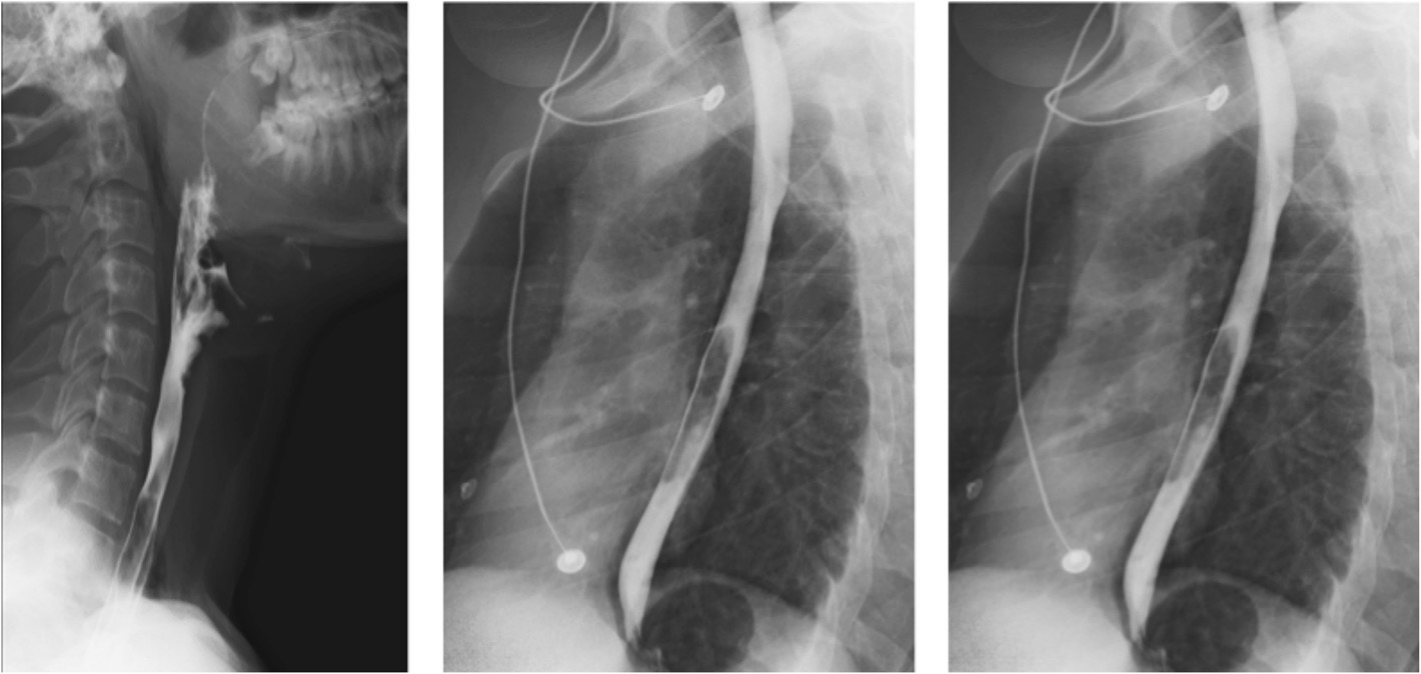
Barium swallow fluoroscopy test showing no esophageal rupture

Results from baseline blood tests, including arterial blood gases, were unremarkable and an electrocardiogram showed a normal sinus rhythm with no arrhythmias or segmental changes. A plain posterioranterior chest radiograph revealed subtle pneumomediastinum and soft tissue emphysema at the base of his neck with no associated pneumothorax (Fig. 1). A plain radiograph of his neck demonstrated extensive subcutaneous emphysema (Fig. 2). Computed tomography of his neck and chest was performed, and confirmed the above radiographic findings (Figs. 3 and 4). No additional sequelae of cocaine use, such as granulomas, emphysema, or bullae, were seen on pulmonary imaging. In order to rule out any esophageal involvement, our patient was transferred to a tertiary center for a barium swallow fluoroscopy study, which was normal (Fig. 5).

He was admitted under the care of the cardiothoracic surgery team at the tertiary center. He was managed conservatively and observed for a further 24 hours, during which the subcutaneous emphysema gradually improved although did not resolve completely. He was discharged home after a total of 48 hours in hospital. He was followed-up in the community by his general practitioner and was found to be well 3 months later.

## Discussion

Subcutaneous emphysema is a very rare condition that is easily identified clinically by palpable crepitations. Whilst it can occur in any part of the body, it is more common in the head and neck because of the proximity of the airway. The etiology is normally iatrogenic or traumatic but there are reported cases of spontaneous subcutaneous emphysema [2, 3]. In our case, the spontaneous subcutaneous emphysema was associated with pneumomediastinum, which is also known as Hamman’s syndrome, a rare condition named after Louis Virgil Hamman (1877–1946), who first described it in 1939 [4, 5]. Pneumomediastinum, which is free air in the mediastinum, is a rare complication of cocaine use and there are a number of reported cases of pneumomediastinum in patients who smoke cocaine, particularly in patient’s who do so on a regular basis [1, 6, 7]. However, there are only a few reported cases of pneumomediastinum after nasal insufflation of cocaine, which makes our case unusual [2, 3].

The pathogenesis of subcutaneous emphysema and pneumomediastinum following cocaine inhalation is thought to be primarily a result of barotrauma [7, 8]. It is caused by increased intra-alveolar pressure and the development of a pressure gradient between the alveoli and vasculature surrounding them [8]. Negative pressure is formed during forced inspiration with a closed mouth and nose (Müller’s manoeuver) and a positive pressure gradient is formed with breath holding (Valsalva manoeuver) [8]. The deliberate production of these maneuvers is thought to maximize the absorption and effect of cocaine by increasing the intrathoracic pressure, thereby increasing the diffusion of the drug across the alveolar membrane into the bloodstream. However, this may lead to alveolar rupture, resulting in air escaping into the mediastinum and fascial planes of the neck [5, 7, 8]. This is presumed to be the mechanism causing subcutaneous emphysema and pneumomediastinum after nasal insufflation of cocaine as well, although data to support this are insufficient [2, 3]. There is also a suggestion that cocaine has direct toxic effects on lung tissue, which cause alveolar damage and hemorrhage that make rupture more likely [6]. This is thought to be more likely in individuals who smoke large quantities of cocaine and/or smoke more frequently: neither scenario applied to our patient [6]. This could explain why there were no other pulmonary features of illicit drug use such as granulomas or fibrosis seen on radiological imaging. Other clinical symptoms and signs of spontaneous emphysema or pneumomediastinum include chest pain, cough, dyspnea, dysphonia, and throat or jaw pain [7].

In practice, spontaneous emphysema and pneumomediastinum are generally benign and self-limiting, requiring only conservative management with careful observation for respiratory compromise. However, it is crucial to consider the potential serious complications and to distinguish this from secondary pneumomediastinum and subcutaneous emphysema. Complications and underlying etiologies associated with these conditions include airway compression, pneumopericardium, esophageal tear, and tracheobronchial rupture. Hence, investigations to rule these out should be undertaken.

## Conclusion

Spontaneous subcutaneous emphysema and pneumomediastinum can usually be managed conservatively without any surgical intervention. However, appropriate investigations are recommended to exclude serious pathology such as esophageal perforation or pneumopericardium. Although rare, the presence of pneumomediastinum should be considered in patients who present with chest pain after cocaine use.

## Consent

Written informed consent was obtained from the patient for publication of this case report and accompanying images. A copy of the written consent is available for review by the Editor-in-Chief of this journal.
